# Gene Amplification-Associated Overexpression of the Selenoprotein tRNA Enzyme TRIT1 Confers Sensitivity to Arsenic Trioxide in Small-Cell Lung Cancer

**DOI:** 10.3390/cancers13081869

**Published:** 2021-04-14

**Authors:** Laia Coll-SanMartin, Veronica Davalos, David Piñeyro, Margalida Rosselló-Tortella, Alberto Bueno-Costa, Fernando Setien, Alberto Villanueva, Isabel Granada, Neus Ruiz-Xiviller, Annika Kotter, Mark Helm, Jun Yokota, Reika Kawabata-Iwakawa, Takashi Kohno, Manel Esteller

**Affiliations:** 1 Josep Carreras Leukaemia Research Institute (IJC), 08916 Barcelona, Spain; lcoll@carrerasresearch.org (L.C.-S.); vdavalos@carrerasresearch.org (V.D.); dpineyro@carrerasresearch.org (D.P.); mrossello@carrerasresearch.org (M.R.-T.); abueno@carrerasresearch.org (A.B.-C.); fsetien@carrerasresearch.org (F.S.); igranada@iconcologia.net (I.G.); nruiz@iconcologia.net (N.R.-X.); 2 Centro de Investigación Biomédica en Red Cáncer (CIBERONC), 28029 Madrid, Spain; 3 Germans Trias i Pujol Health Science Research Institute (IGTP), 08916 Barcelona, Spain; 4 Group of Chemoresistance and Predictive Factors, Subprogram Against Cancer Therapeutic Resistance (ProCURE), Oncobell Program, IDIBELL, Institut Català d’Oncologia (ICO), L’Hospitalet del Llobregat, 08908 Barcelona, Spain; avillanueva@iconcologia.net; 5 Cytogenetics Platform, Hematology Laboratory Service, Institut Català d’Oncologia (ICO)-Hospital Germans Trias i Pujol (IGTP), 08916 Barcelona, Spain; 6 Institute of Pharmaceutical and Biomedical Sciences, Johannes Gutenberg-Universität Mainz, 55128 Mainz, Germany; akotter@uni-mainz.de (A.K.); mhelm@uni-mainz.de (M.H.); 7 Division of Genome Biology, National Cancer Center Research Institute, Tokyo 104-0045, Japan; jyokota-catv@j05.itscom.net (J.Y.); r.kawabata@gunma-u.ac.jp (R.K.-I.); tkkohno@ncc.go.jp (T.K.); 8 Division of Integrated Oncology Research, Gunma University Initiative for Advanced Research, Gunma 371-8511, Japan; 9 Division of Translational Genomics, Exploratory Oncology Research and Clinical Trial Center, National Cancer Center, Tokyo 104-0045, Japan; 10 Institucio Catalana de Recerca i Estudis Avançats (ICREA), 08010 Barcelona, Spain; 11 Physiological Sciences Department, School of Medicine and Health Sciences, University of Barcelona (UB), 08036 Barcelona, Spain

**Keywords:** small-cell lung cancer, transfer RNA, RNA modifications, TRIT1, gene amplification, selenoproteins

## Abstract

**Simple Summary:**

Small-cell lung cancer accounts for approximately 13% of all new lung cancer diagnoses, but in contrast to non-small-cell lung cancer, the implementation of targeted treatments in small-cell lung cancer has been limited, with little improvement in the clinical outcome in the last several decades. Exploring new pathways for targeted therapy, we have observed that extra-copies of the tRNA modifier TRIT1, involved in the translation of selenoproteins, confers sensitivity to arsenic trioxide in small-cell lung cancer. This finding could open a new therapeutic niche for a tumor type with such a dismal clinical course.

**Abstract:**

The alteration of RNA modification patterns is emerging as a common feature of human malignancies. If these changes affect key RNA molecules for mRNA translation, such as transfer RNA, they can have important consequences for cell transformation. TRIT1 is the enzyme responsible for the hypermodification of adenosine 37 in the anticodon region of human tRNAs containing serine and selenocysteine. Herein, we show that TRIT1 undergoes gene amplification-associated overexpression in cancer cell lines and primary samples of small-cell lung cancer. From growth and functional standpoints, the induced depletion of TRIT1 expression in amplified cells reduces their tumorigenic potential and downregulates the selenoprotein transcripts. We observed that TRIT1-amplified cells are sensitive to arsenic trioxide, a compound that regulates selenoproteins, whereas reduction of TRIT1 levels confers loss of sensitivity to the drug. Overall, our results indicate a role for TRIT1 as a small-cell lung cancer-relevant gene that, when undergoing gene amplification-associated activation, can be targeted with the differentiation agent arsenic trioxide.

## 1. Introduction

Altered RNA and protein patterns are a feature of human tumors. These aberrant expression profiles may arise by myriad mechanisms. One interesting possibility is that the dysregulated cancer transcriptome and proteome are linked to aberrant RNA modification landscapes. RNA molecules exhibit chemically modified nucleosides that together form the denominated epitranscriptome [1,2,3]. More than 100 differentially modified nucleotides, affecting RNA stability, targeting, translation, and other activities have been reported. An emerging body of data in cancer biology indicates that significant changes occur in the epitranscriptome with the appearance of genetic and epigenetic defects in RNA-modifier genes [1,2,3]. RNA modifications are particularly relevant in transfer RNA molecules (tRNAs). These affect protein translation function at several levels, including amino acid loading, wobbling or translation efficiency, and translational fidelity [4,5]. Importantly, tRNA modifications and tRNA modifier enzymes are disrupted in a variety of human diseases, including cancer [6,7,8].

One example of a chemical modification of tRNA with a putative role in tumorigenesis is the appearance of hypermodified adenosines at position A37, adjacent to the anticodon of serine (tRNA^Ser^) and selenocysteine (tRNA^[Ser]Sec^) tRNAs [9,10,11]. The A37 position in the anticodon loop is modified to N^6^-isopentenyladenosine (i^6^A) by the tRNA isopentenyltransferase 1 (TRIT1) enzyme [10,11,12] and can be further hypermodified by CDK5RAP1, which converts i^6^A to 2-methylthio-N^6^-isopentenyl adenosine (ms^2^i^6^A) [12,13,14,15,16]. The correct hypermodification of A37 is essential for the specific activity of its associated tRNAs, which regulate translation fidelity and efficiency [17]. A potential link to cancer involves the effect of the hypermodified A37 on tRNA^[Ser]Sec^ functionality and, thereby, on selenoprotein synthesis [18]. Selenium-containing proteins fulfil pleiotropic roles in carcinogenesis, such as the detoxification of reactive oxygen species, the modulation of calcium homeostasis and unfolded protein responses in the endoplasmic reticulum [19,20], and the reduction in selenoprotein expression due to the impairment of isopentenylated tRNAs [9,21]. These findings prompted us to investigate the presence of genetic and epigenetic defects in the A37-modifying enzymes TRIT1 and CDK5RAP1 in human tumors. Herein, we describe how TRIT1 undergoes gene amplification-associated overexpression in cancer cell lines and primary samples of small-cell lung cancer, giving rise to growth-inhibitory sensitivity to arsenic trioxide.

## 2. Materials and Methods

### 2.1. Human Cell Lines

For this study, NCI-H82 (American Type Culture Collection, ATCC), DMS-273 (Sigma-Aldrich, St. Louis, MO, USA) and HCC-33 (Leibniz Institute DSMZ, German Collection of Microorganisms and Cell Cultures) human small-cell lung cancer (SCLC) cell lines, and HEK293 (ATCC) human embryonic kidney cell line were used. NCI-H82, HCC-33, and HEK293 cell lines were cultured with Roswell Park Memorial Institute (RPMI1640, Gibco, Waltham, MA, USA) medium and, DMS-273 cell lines were cultured with Waymouth (Gibco, Waltham, MA, USA) medium. Culture medium was completed with 10% fetal bovine serum (FBS, Gibco, Waltham, MA, USA) and 1% of penicillin/streptomycin (Invitrogen, Carlsbad, CA, USA). Cells were cultured at 37 °C with 5% (*v*/*v*) of CO2. All cell lines tested negative for mycoplasma.

### 2.2. Human Biological Samples

DNAs isolated from tumor samples of 39 SCLC patients obtained between 1975 and 2010 at the National Cancer Center Hospital/National Cancer Center Biobank (Tokyo, Japan), Saitama Medical University (Saitama, Japan), and the University of Tsukuba (Ibaraki, Japan) were used in this study. The study was approved by the corresponding Institutional Review Boards.

### 2.3. Fluorescence In Situ Hybridization (FISH)

The UCSC genome browser was used to select the 1p34.2 region probe RP11-613D14 for TRIT1 detection. Bacterial artificial chromosome (BAC) clones were obtained from the BACPAC Resources Center at the Children’s Hospital Oakland Research Institute (Oakland, CA, USA). Probe was labeled with Spectrum Red dUTP (Abbott, Chicago, IL, USA) and CGH Nick Translation Kit with control DNA (MPE 600, Abbott, Chicago, IL, USA). FISH was performed on cells fixed in Carnoy’s solution. The sample and probe were codenatured by heating slides on a hotplate at 75 °C for 2 min. After that, they were hybridized with 5 µL of handmade probe mixture (BAC RP11-613D14, 1p34.2/TRIT1) or control (D-5099-100-OG, MetaSystems, 1p32.3) and incubated in a humidified chamber at 37 °C overnight. Post-hybridization washes of hybridized slides were performed first with 0.4X SSC (pH 7.0) at 72 °C for 2 min followed by a wash in 2X SSC, 0.05% Tween-20 (pH 7.0) at room temperature for 30 s. Finally, slides were counterstained with DAPI and analyzed under a fluorescent microscope (NIKON, Eclipse E400).

### 2.4. Multiplex Ligation-Dependent Probe Amplification (MLPA)

DNA samples from SCLC patients and SCLC cell lines were sent to qGenomics (Spain) for MLPA analysis. Two probes for exons 4 and 9 of the TRIT1 gene were designed. RAC1 (exon 6), TBCK (exon 15), TRPM7 (exons 17 and 18), and TRIP12 (exons 3 and 11) probes were used as references.

### 2.5. TRIT1 Short-Hairpin RNA

Lentiviral plasmids for TRIT1 human shRNA (TL300819-C, Origene, Rockville, MD, USA) and scrambled shRNA (TR30021, Origene, Rockville, MD, USA), both cloned in pGFP-C-shLenti vector, were used. To obtain the lentiviral particles, 10 μg of plasmid were mixed with 7.5 μg of ps-PAX2 and 2.5 μg of PMD2.G plasmid (Addgene, Watertown, MA, USA), using jetPRIME^®^ Transfection Reagent (Polyplus Transfection, New York, NY, USA). The transfection mix was added to HEK293 cells at 80% confluence. After 72 h, medium with high-titer lentiviral particles was 0.45 μm-filtered and DMS-273 cells were cultured in virus containing medium for 24 h. After five passages, green fluorescent cells were sorted by fluorescence-activated single cell sorting (FACS).

### 2.6. Western Blot

Cells were collected by scraping after two washes with cold PBS. Cell pellets were resuspended in RIPA (0.1% SDS, 50 mM Tris-HCl [pH 7.4], 150 mM NaCl, 1 mM EDTA, 1% NP-40, 12.23 mM deoxycholic acid) containing protease and the cOmplete™ (Roche, Basel, Switzerland) phosphatase inhibitor cocktail, then incubated for 20 min on ice. The tubes were centrifuged for 5 min at 13,000 rpm, and the supernatant was collected. RIPA extracts were quantified using the Pierce™ BCA Protein Assay Kit (Thermo Scientific, Waltham, MA, USA). Each sample was diluted 1:6 in water and 10 μL of each sample were loaded in triplicate into a 96-well plate. A standard curve of albumin was performed to determine protein concentration. Kit reagents were prepared and loaded according to the manufacturer’s instructions. After 30 min, the 96-well plate was read at 562 nm. TRIT1 protein expression was analyzed by Western blot. SDS-PAGE was performed on acrylamide gels with an acrylamide percentage of 12%. Membranes were blocked with 5% skimmed milk (BD Difco, 232100) in 0.1% Tween-20 in PBS (PBS-Tween) for 1 h with shaking. Membranes were incubated overnight at 4 °C with TRIT1 antibody (NBP2-20727, Novus Biologicals, 1:1000 dilution). Afterwards, membranes were washed three times with PBS-Tween and incubated with anti-rabbit HRP-conjugated secondary antibody (A0545, Sigma Aldrich, St. Louis, MO, USA, 1:10,000 dilution) for 1 h with shaking. Membranes were washed three times with PBS-Tween and developed using Luminata HRP-substrates (Millipore, Burlington, MA, USA). As a loading control, membranes were incubated with β-actin (Actin) HRP-conjugated antibody (A3854, Sigma Aldrich, St. Louis, MO, USA, 1:5000).

### 2.7. N^6^-Isopentenyladenosine (I^6^A) Quantification

Cells were lysed using TRI Reagent (T9424, Sigma Aldrich, St. Louis, MO, USA) and phase separation was performed with 1-bromo-3-chloropropane. The mixture was centrifuged in order to obtain the aqueous phase and to precipitate the RNA with 2-isopropanol. RNA pellets were washed with ethanol, dried, and resuspended in water. The RNA concentration was measured with a Nanodrop (ThermoFisher, Waltham, MA, USA) spectrophotometer. Modification status of A37 was finally determined by liquid chromatography–mass spectrometry (LC/MS).

### 2.8. Murine Models

To analyze tumor growth, DMS-273 SCR or shTRIT1 cells were subcutaneously injected into the flanks of five-week-old athymic nu/nu mice (Envigo Laboratories). Three million cells in 50% Matrigel (354234, BD Biosciences) for each condition were injected into nine mice per group. Tumor development was monitored every 4–6 days, and tumor size (width: W and length: L) was measured with a caliper to calculate the volume (V = π/6 × L × W^2^). Animals were sacrificed 20 days after injection.

For the arsenic trioxide treatment, DMS-273 SCR or shTRIT1 cells were subcutaneously injected in the mouse flanks as described above. Ten days after injection, the animals were randomized in two groups and treated with vehicle (0.015 N NaOH in saline) or Arsenic Trioxide (A1010, Sigma-Aldrich, St. Louis, MO, USA) diluted in 0.015 N NaOH. Drug was administered by intraperitoneal injection at 5 mg/kg dosage following a schedule of 5 days ON/2OFF for three consecutive weeks. Tumor growth was monitored and measured as described above and animals were sacrificed 28 days after cell-line inoculation. All mouse experiments were approved by the Institutional Animal Care Committee of Bellvitge Biomedical Research Institute (IDIBELL) and performed in accordance with the guidelines of the International Guiding Principles for Biomedical Research Involving Animals, developed for the Council for International Organizations of Medical Sciences (CIOMS). The IDIBELL animal facility is accredited by the AAALAC (Association for Assessment and Accreditation of Laboratory Animal Care International, Unit 1155) since 2006 and works according to the European and National Legislation (CEE/86/609, RD1201/2005, RD214/1997). Our experimental procedure (9111) was revised and approved by local Government of Generalitat de Catalunya.

### 2.9. Drug-Dose Response Assay

After determining the optimal number, 20,000 cells were seeded onto 96-well plates. After overnight incubation, cells were treated with decreasing concentrations of arsenic trioxide (A1010, Sigma-Aldrich, St. Louis, MO, USA) or cisplatin (cis-diammineplatinum(II)dichloride, P4394, Sigma-Aldrich, St. Louis, MO, USA) in order to calculate the half-maximal inhibitory concentration (IC50). After 48 h, MTT (3-(4,5-dimethyl-2-thiazolyl)-2,5-diphenyl-2H-tetrazolium bromide, Sigma-Aldrich, M2128-10G) reagent was added. After 3 h of incubation at 37 °C, cells were lysed for about 20 h with MTT lysis buffer (50% N-N dimethylformamide, 20% sodium dodecyl sulfate, 2.5% glacial acetic acid, 2.1% 1 N HCl, at pH 4.7), and absorbance was read at 560 nm with an optical spectrometer. IC50 was determined with GraphPad Prism (Version 5 for Windows, GraphPad Software, La Jolla, CA, USA).

### 2.10. RNA Sequencing

Total RNA from DMS-273 SCR and shTRIT1 cells was extracted using a Maxwell RSC device (Promega, Madison, WI, USA). Then, 5 μg of total RNA from three biological replicates from each sample were used for RNA sequencing (RNA-seq). The RNA-seq libraries were prepared from total RNA with TruSeq^®^Stranded mRNA LT Sample Prep Kit (Illumina, San Diego, CA, USA). Each library was sequenced using a TruSeq SBS Kit v4-HS, in paired-end mode with a read length of 2 × 76 + 8 + 8bp. We obtained ~500 million paired-end reads in a fraction of a sequencing lane on HiSeq2500 (Illumina, San Diego, CA, USA), following the manufacturer’s protocol. Raw reads were quality assessed and preprocessed using FASTQC (version 0.11.7, Babraham Bioinformatics, Babraham Institute, Cambridge, UK) and Trimmomatic (version 0.36, The Usadel Lab, Aachen University, Aachen, Germany) software. Differential expression analysis was performed using DESeq2 Bioconductor package (v1.18.1), in R (v3.4.3). Gene annotations were extracted from GENECODE (v35). Gene ontology (GO) biological processes for the downregulated genes with a log2-fold change >|1| and a false discovery rate (FDR) adjusted *p*-value < 0.05 included in the GSEA signature database were used to perform an enrichment analysis.

### 2.11. Statistical Analysis

Associations between variables were assessed by Student’s t test and Wilcoxon rank sum test. Statistical analyses were carried out with GraphPad Prism 5 for Windows (La Jolla, CA, USA). Values of *p* < 0.05 were considered statistically significant (* *p* < 0.05; ** *p* < 0.01; *** *p* < 0.001). All statistical tests were two-sided. The values corresponding to TRIT1 copy number and expression in cell lines were obtained from the Broad Institute Cancer Cell Line Encyclopedia (CCLE) (https://depmap.org/portal/download/ Public Release 20Q4, accessed on 13 January 2021).

## 3. Results

### 3.1. Detection of TRIT1 Gene Amplification-Associated Overexpression in Small-Cell Lung Cancer Cell Lines

**Figure 1:**
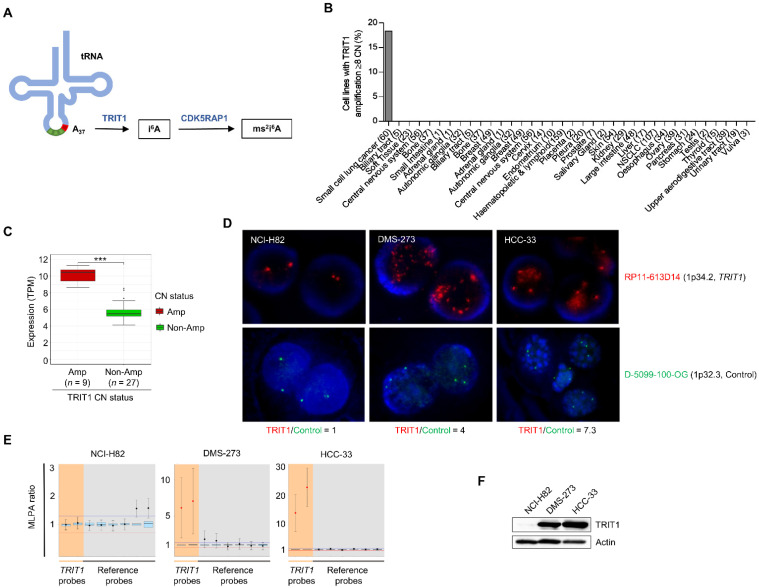
Determination of TRIT1 gene amplification and RNA and protein overexpression in small-cell lung cancer cell lines. (**A**) Schematic representation of adenosine derivatives synthesis at position A37 of human tRNA. The enzymes catalyzing these reactions are shown in blue. Abbreviations are: A, adenosine; i^6^A, N^6^-isopentenyladenosine; ms^2^i^6^A, 2-methylthio-N^6^-isopentenyladenosine. (**B**) Frequency of TRIT1 gene amplification in the panel of cancer cell lines. (**C**) TRIT1 gene amplification was significantly associated with high levels of the TRIT1 transcript in the small-cell lung cancer cell lines for which expression patterns were available (*n* = 36). Non-Amp, non-amplified; Amp, amplified. TPM, transcripts per million. *p*-value obtained by Wilcoxon rank sum test. *** *p* < 0.001. (**D**) Fluorescence in situ hybridization for the TRIT1 gene. The UCSC genome browser was used to select the bacterial artificial chromosome (BAC) clone spanning the 1p34.2 region for the TRIT1 gene: RP11-613D14. The BAC was obtained from the BACPAC Resource Center at the Children’s Hospital Oakland Research Institute (Oakland, CA, USA). TRIT1 probe was labeled with Red dUTP (Abbott, Wiesbaden, Germany), using a CGH Nick Translation Reagent Kit (Abbott Molecular Inc., Des Plaines, IL, USA). The samples were counterstained with DAPI and analyzed under a fluorescent microscope (NIKON, Eclipse E400). Gene amplification was found in the interphases of DMS-273 and HCC-33. The D-5099-100-OG probe (1p32.3, MetaSystems) was used as control. TRIT1/Control ratios are shown. (**E**) Multiplex ligation-dependent probe amplification (MLPA) assay. Probe mixes contained two probes for exons 4 and 9 of the TRIT1 gene (in orange). Six reference probes were also included (in grey). Values greater than 2 (two copies, corresponding to MLPA ratio of 1) were considered to indicate the presence of extra copies. DMS-273 and HCC-33 cell lines showed TRIT1 gene amplification, whilst NCI-H82 is shown as an example of TRIT1 two copy number cells. (**F**) TRIT1 expression levels in gene-unamplified (NCI-H82) and amplified (DMS-273 and HCC-33) cancer cell lines determined by Western blot analysis. Actin is shown as the loading control. The uncropped Western blots have been shown in Figure S3.

To find tumor-associated genetic and epigenetic changes in the A37-modifying enzymes TRIT1 and CDK5RAP1 (Figure 1A), we data-mined a collection of about 1000 human cancer cell lines in which the transcriptome, DNA methylation landscape, exome sequence, and gene copy number were available [22,23]. The available multiomics data did not reveal the presence of TRIT1 or CDK5RAP1 mutations, deletions, or promoter hypermethylation events in the analyzed cell lines (Supplementary Materials Dataset S1). However, the TRIT1 gene was found to be amplified in 18.3% (11 of 60) of small-cell lung cancer cell lines (Figure 1B and Supplementary Materials Dataset S1). Gene amplification did not occur in the other tumor types. Data-mining of the available RNA expression patterns in the described set of small-cell lung cancer cell lines [23] demonstrated that TRIT1 gene amplification was associated with the overexpression of its mRNA (Figure 1C). Related to the genetic context, small-cell lung cancer is highly mutated at the RB1 and TP53 tumor suppressor genes. We observed that 82% (9 of 11) and 100% (11 of 11) of the TRIT1 amplified cell lines showed RB1 and TP53 mutations, respectively; an indistinguishable distribution to the one found in the non-amplified cell lines (Fisher’s exact test, *p* = 0.4781 and *p* = 1, respectively).

Having found the above-described TRIT1 copy-number patterns, we proceeded to study TRIT1 gene amplification and its associated expression in detail in the small-cell cancer lines NCI-H82, DMS-273 and HCC-33 that were included in the characterized set [22,23]. Using fluorescence in situ hybridization (FISH), we observed the presence of TRIT1 gene amplification in the DMS-273 and HCC-33 cell lines, whereas NCI-H82 showed the expected two copies of the genes (Figure 1D). These data confirmed the copy-number results obtained from the whole exome sequencing (WES) data-mining (Supplementary Materials Dataset S1). The use of multiplex ligation-dependent probe amplification (MLPA) technology confirmed the occurrence of TRIT1 gene amplification in DMS-273 and HCC-33 cells and its absence in NCI-H82 cells (Figure 1E). Most notably, the presence of TRIT1 gene amplification was associated with the overexpression of the TRIT1 protein in the DMS-273 and HCC-33 small-cell lung cancer cell lines determined by western-blot (Figure 1F) and immunocytochemistry (Figure S1).

### 3.2. Cellular and Molecular Effects of TRIT1 Depletion in Gene Amplified Small-Cell Lung Cancer Cells

**Figure 2:**
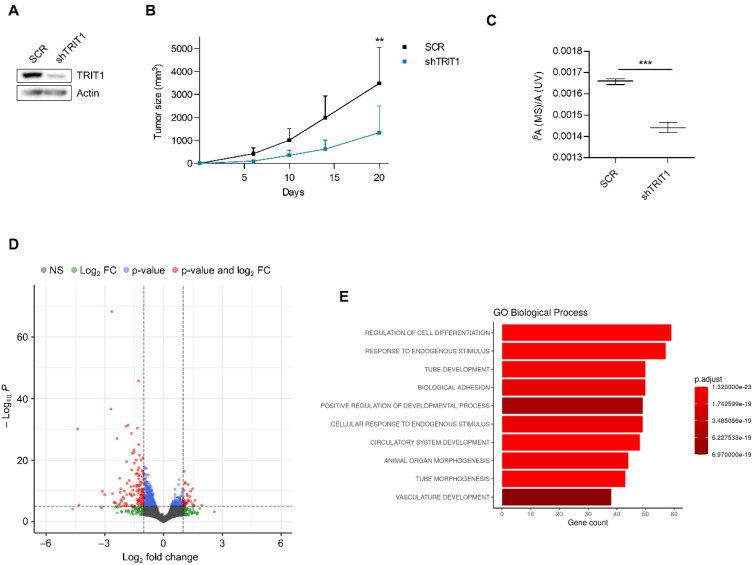
Effect of TRIT1 depletion on tumor growth and the RNA transcriptome of small-cell lung cancer. (**A**) Stable downregulation of the TRIT1 gene by short hairpin RNA in the small-cell lung cancer cell line DMS-273 (shTRIT1) determined by western blot analysis. SCR, scramble shRNA. (**B**) Effect of TRIT1 shRNA-mediated depletion on the growth of subcutaneous tumors in nude mice derived from DMS-273 cells (amplified and overexpressing TRIT1). There was a significant reduction in tumor volume in the TRIT1 shRNA-depleted cells. Data are summarized as the mean and standard deviation (*n* = 9). Student’s *t* test, ** *p* < 0.01. (**C**) Nucleoside analysis of tRNAs by LC/MS showing that shRNA-mediated depletion of TRIT1 in DMS-273 cells induces the depletion of the i^6^A-modified nucleoside. Student’s *t* test, *** *p* = 0.0002. (**D**) Volcano plot summarizing the results of the RNA-seq experiment to find mRNAs differentially expressed in TRIT1 shRNA-depleted DMS-273 cells compared with scramble-shRNA DMS-273 cells. (**E**) Gene ontology (GO) analysis of Biological Process categories in the transcripts downregulated on TRIT1 depletion in DMS-273 cells shows the GO Biological Process category “regulation of cell differentiation” to be the most highly enriched.

Once we had demonstrated the existence of TRIT1 gene amplification-associated overexpression in small-cell lung cancer cell lines, we interrogated its role in in vivo tumor growth and its impact on i^6^A-tRNA-associated activity. To these ends, we downregulated the expression of TRIT1 in the gene-amplified DMS-273 cell line. The efficient depletion of TRIT1 by the short hairpin RNA approach was confirmed by Western blot (Figure 2A). We first characterized the capacity of shRNA-depleted TRIT1 (shTRIT1) DMS-273 cells to form subcutaneous tumors in nude mice compared with shRNA-scramble (SCR) cells. The downregulation of TRIT1 levels in these small-cell lung tumors reduced their growth in comparison with shRNA-scramble DMS-273-derived tumors, as demonstrated by the continuous measurement of the tumor volume (Figure 2B).

Following the demonstration of how TRIT1 loss impaired tumor growth, we analyzed its effect on the chemical modification status of A37 using tRNA-associated liquid chromatography–mass spectrometry (LC/MS) [24]. We found that shRNA-mediated downregulation of TRIT1 in DMS-273 cells induced a decrease in levels of the i^6^A nucleoside (Figure 2C), as expected given the known function of the enzyme [10,11,12]. We then wondered whether the depletion of i^6^A content in these TRIT1 shRNA-downregulated cells had any impact on the underlying transcriptome profile due to the deficient hypermodification of the tRNAs containing this particular nucleoside. To address this, we carried out RNA-seq in the shRNA-scramble DMS-273 cells harboring the amplified TRIT1 gene and cells derived from them that exhibited shRNA-mediated depletion of the TRIT1 transcript. DMS-273 shRNA-scramble and shRNA TRIT1 RNA-seq data have been deposited in the Sequence Read Archive repository (SRA) under project code PRJNA692378. We found that inducing TRIT1 depletion in the cells featuring gene amplification altered the levels of 4510 mRNAs (Table S1). This mostly took the form of downregulation, which occurred in 75.6% (3409 of 4510) of mRNAs (Figure 2D). Importantly, of the 25 proteins containing selenocysteine described in human cells, seven were found to be downregulated upon depletion of TRIT1 (hypergeometric test, *p* = 0.00238), reinforcing the concept that defects in isopentenylated tRNAs reduce selenoprotein expression [9,21]. The downregulated selenoproteins were GPX1, GPX3, GPX4, SELENON, SELENOP, SELENOW, and TXNRD1. Gene set enrichment analysis (GSEA) assessed by gene ontology (GO) signature collections in the group of downregulated RNAs demonstrated as the first category an overrepresentation of the GO biological process “regulation of cell differentiation” (Figure 2E).

### 3.3. Occurrence of TRIT1 Gene Amplification in Small-Cell Lung Cancer Patients and In Vitro and In Vivo Sensitivity to Arsenic Trioxide

**Figure 3:**
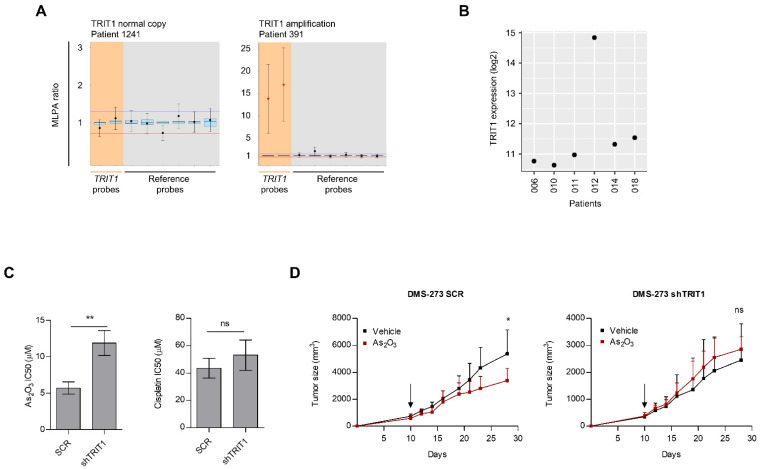
TRIT1 gene amplification in primary small-cell lung cancer patients and response to arsenic trioxide. (**A**) MLPA assay of primary small-cell lung cancer samples. Probe mixes contained two probes for exons 4 and 9 of the TRIT1 gene (in orange). Six reference probes were also included (in grey). Values greater than 2 (two copies, corresponding to MLPA ratio of 1) were considered to indicate the presence of extra copies. Patient 1241 is shown as example of a TRIT1 two copy number case, whilst patient 391 shows TRIT1 gene amplification. (**B**) TRIT1 RNA expression levels derived from Affymetrix U133Plus2.0 microarray data in six primary small-cell lung cancer samples where TRIT1 copy number was determined. The only patient that exhibited TRIT1 gene amplification (#012) showed the highest TRIT1 expression level. Two copies of TRIT1 were observed in the 006, 010, 011, 014 and 018 samples. (**C**) IC50 determination by MTT assay. TRIT1 shRNA-depleted DMS-273 cells were significantly less sensitive to the antiproliferative effect of arsenic trioxide than were the shRNA scramble-transfected cells harboring TRIT1 gene amplification-associated overexpression. TRIT1 shRNA-mediated depletion did not affect sensitivity to cisplatin. Student’s *t* test, ** *p* < 0.01; ns, non-significant. (**D**) shRNA scramble (SCR, left panel) and TRIT1 shRNA-depleted (shTRIT1, right panel) DMS-273 cells were injected into the flanks of nude mice to form subcutaneous tumors. Tumor volume over time according to treatment conditions, vehicle (black lines) vs. arsenic trioxide-treated group (red lines) are shown. Black arrow indicates the time at which the mice were randomized and started to be treated with arsenic trioxide or vehicle. P values are those corresponding to Student’s *t* tests. Means and standard deviations (bars) are illustrated. Tumors derived from shRNA scramble-transfected DMS-273 cells were sensitive to the growth inhibition effect of arsenic trioxide (left panel), whilst TRIT1 shRNA-mediated depletion eliminates the enhanced sensitivity to arsenic trioxide, the tumor size reduction effect being similar to that obtained with the vehicle treatment (right panel). * *p* < 0.05; ns, non-significant.

The occurrence of TRIT1 gene amplification was not an event confined to in vitro-grown small-cell lung cancer cell lines, but was also observed in primary tumor samples. We identified TRIT1 gene amplification in 10.3% (4/39) of primary small-cell lung cancer cases. The clinicopathological features are summarized in Table 1. Examples of the MLPA assay in small-cell lung cancer patients are shown in Figure 3A. The presence of extra copies of TRIT1 was not associated with patient gender, smoking status, or clinical stage (Table 1). Stage IV, which accounts for 85% of cases at diagnosis, was not enriched in our cohort, being a potential limitation of our study. The distribution of RB1 and TP53 mutations [25] was not different between TRIT1 amplified and non-amplified cases (Fisher’s exact test, *p* = 1). TRIT1 gene amplification was not associated with overall survival (hazard ratio = 0.625; *p* = 0.449; 95% CI = 0.185–2.113; log-rank test, *p* = 0.445). Expression microarray data were available for six of these small-cell lung cancer cases [26]. For this subset of six samples, we observed that the only patient with TRIT1 gene amplification showed the highest TRIT1 expression levels, whereas the remaining five cases without TRIT1 extra-copies showed lower expression (Figure 3B). We expanded these numbers by datamining a collection of 68 small-cell lung cancer cases where SNP microarray data are available [27]. TRIT1 gene amplification was observed in 14.7% (10/68) of primary small-cell lung cancer cases in this dataset. Expression microarray data were available for 23 cases of the described cohort [27]. For this subset, the presence of TRIT1 gene amplification (observed in three cases) was correlated with higher TRIT1 expression levels (Spearman’s correlation test, *p* = 0.05). The distribution of RB1 and TP53 mutations [28] was not different between TRIT1 amplified and non-amplified cases (Fisher’s exact test, *p* = 1). We also wondered about the distribution of TRIT1 gene amplification according to the small-cell lung cancer molecular subtypes ASCL1, NEUROD1, POU2F3, and YAP1 [29]. Among the 23 cases available for expression patterns, we found that the three cases with TRIT1 gene amplification occurred all in the ASCL1 subtype.

Since there are few therapeutic options for treating this malignancy and little evidence that they have improved outcomes in recent years [30,31], we wondered whether the copy number gain of TRIT1 is associated with an increased sensitivity to antitumor drugs. Arsenic trioxide could be one such candidate compound [32,33]. It is highly effective in the treatment of acute promyelocytic leukemia (APL) [34] and has shown considerable therapeutic potential for treating other malignancies [32,33], including lung cancer [35]. The effect of arsenic trioxide in APL involves inducing differentiation of the leukemia cells [34], the “differentiation” category being the most overrepresented in the GO analysis of TRIT1-disrupted cells (Figure 2E). Most significantly, the downregulation of selenoproteins, observed in TRIT1-depleted cells (Table S1), induces loss of sensitivity to arsenic trioxide-mediated cytotoxicity [36], and arsenic trioxide inhibits selenoprotein activity [37] and synthesis [38]. In this regard, there is also an emerging interest in targeting selenoproteins for therapeutic purposes [39]. All these observations prompted us to assess whether TRIT1 expression levels were associated with arsenic trioxide sensitivity. Using the model of TRIT1 shRNA-mediated downregulation and the calculation of IC50 values according to the MTT assay, we found that DMS-273 scramble shRNA cells overexpressing the TRIT1 gene were significantly more sensitive to the growth-inhibitory effect mediated by arsenic trioxide than were TRIT1-depleted cells (Figure 3C). The effect was specific to the arsenic trioxide drug and we found no difference in the sensitivity to cisplatin between the two cell lines (Figure 3C), a chemotherapy agent commonly used in the clinical treatment of small-cell lung cancer. We extended the in vitro cell viability experiments to the in vivo mouse model described above, and found that DMS-273 scramble shRNA-derived tumors that harbored gene amplification-associated overexpression of TRIT1 responded markedly to the administration of arsenic trioxide, whereas the shRNA-mediated depletion of TRIT1 abolished the growth-inhibitory effect of arsenic trioxide, whereby the tumors underwent a similar increase in volume to those of the animals treated with the simple drug vehicle (Figure 3D). Thus, these results suggest that small-cell lung cancers carrying extra copies of TRIT1 are probably responders to arsenic trioxide.

## 4. Discussion

Small-cell lung cancer is an aggressive malignancy in which a majority of patients at first diagnosis already show metastatic disease outside the hemithorax [30,31]. It accounts for approximately 13% of all new lung cancer diagnoses, but in contrast to non-small-cell lung cancer the implementation of targeted treatments in small-cell lung cancer has been limited, with chemotherapy having been the standard therapy for first- and second-line clinical management for several decades [30,31]. One of the main issues is the appearance of chemoresistance at recurrence, a type of sample that was not included in our study. One reason for the slow development of gene-oriented therapeutic approaches is that if there are non-small-cell lung cancer cases with driver oncogenic-activating genetic events (such as in the EGFR, ALK, and ROS genes) [40], small-cell lung cancer exhibits mostly loss-of-function events in its pathogenesis (such as mutations in the tumor suppressor genes RB1 or TP53). These molecular lesions could be targeted by drugs derived from synthetic lethal screenings against these antioncogenes, but there has been little clinical success in this area. As for other human tumor types, it would be best to act on an amplified oncogene with a specific compound. In small-cell lung cancer, we would like to propose that the gene amplification-associated overexpression of the tRNA modifier TRIT1 could be an optimal bona fide target candidate in this context. Although there are no publicly available small compound inhibitors of TRIT1 enzymatic activity, arsenic trioxide, through its direct effects on selenium-containing proteins [36,38], is potentially effective in small-cell lung cancer cells with high levels of TRIT1. The final cellular effects of the agent in cancer cells involve induction of differentiation in the case of its approved use in APL [34] which was the most highly enriched gene ontology category in our TRIT1 shRNA experiment. However, more general mechanisms involving apoptosis and overall cytotoxicity can also be invoked in human malignancies [32,33]. In this regard, arsenic trioxide has several intracellular targets beyond selenoproteins. Interestingly, the expression levels of SOX2, a recognized oncogene in small-cell lung cancer [41], are downregulated upon arsenic trioxide in lung cancer cells [42] and we have also observed downregulation of SOX2 upon TRIT1 shRNA-mediated depletion (Table S1), reinforcing again the link between TRIT1 and arsenic trioxide. For non-small-cell lung cancer, preclinical in vitro data suggest its potential antitumor role [35], but it can be in the setting of small-cell lung cancer with the use of TRIT1 gene amplification as an associated biomarker in which its true therapeutic value for solid malignancies may be revealed. Arsenic trioxide can be administered by intravenous infusion or orally, but it is an optimal candidate for conversion to a biocompatible nanoparticulate form that would reduce the required dose, limiting any adverse effects of the bulk form [32]. Beyond arsenic trioxide, other drugs could also be more active in small-cell lung tumors harboring TRIT1 gene amplification. For example, data-mining the Sanger collection of cancer cell lines where the IC50 values for 265 drugs are available, we observed that small-cell lung cancer cells with TRIT1 extra-copies of TRIT1 were also sensitive to dimethyloxalylglycine (DMOG), a competitive inhibitor against all 2-OG-dependent dioxygenases (Figure S2).

In addition to expanding the role of the epitranscriptome in tumorigenesis [1,2], illustrated here by the described tRNA modification, another aspect of the TRIT1 gene amplification in small-cell lung cancer might have an additional impact on the treatment of this malignancy. The first significant improvement in the clinical outcome of small-cell lung cancer in several decades is the recent addition of immune checkpoint blockade to the therapeutic arsenal against the disease [30,31]. This led the US Food and Drug Administration to approve the use of the anti-PD-L1 inhibitor atezolizumab in combination with carboplatin plus etoposide chemotherapy to treat extensive-stage disease [43]. A significant clinical improvement was subsequently reported for the combination of durvalumab plus platinum-etoposide [44]. These results are very encouraging, although the observed clinical effect has often been modest [43,44]. Thus, it is important to identify the subset of cases that would respond best to the immunotherapy. In this regard, tumor mutational burden (TMB) and PD-L1 expression are proposed as predictive markers of response to immune checkpoint inhibitors (ICIs) in human tumors, including small-cell lung cancer [45,46], although other recent studies have not reported the association of these biomarker with clinical benefit in extensive small-cell lung cancer treated with ICIs [47,48]. Interestingly, the 3′-untranslated region of TRIT1 contains an open reading frame in the amplified region that generates a peptide recognized by cytotoxic T lymphocytes, leading to the lysis of melanoma cells [49]. Thus, a hypothetical role of TRIT1 gene amplification in immunotherapy is worthy of further investigation.

## 5. Conclusions

Overall, our results show that the tRNA modifier gene TRIT1 undergoes gene amplification-associated overexpression in small-cell lung cancer cell lines and primary samples. It is an aberrant genomic lesion that promotes tumor growth and is associated with imbalance of the hypermodified adenosine adjacent to the anticodon of selenocysteine tRNAs, and dysregulates the expression levels of selenoproteins that exert pleiotropic functions in tumorigenesis. In vitro and in vivo data indicated that extra TRIT1 copies confer sensitivity to the differentiating drug arsenic trioxide, providing a new candidate therapeutic niche that merits further research, especially for a tumor type with such a dismal clinical outcome.

## Figures and Tables

**Table 1:** Clinicopathological features of the studied small cell lung cancer patients according to TRIT1 gene amplification status.

Clinical Characteristics	Total (*n* = 39)	TRIT1 Non-Amplified (*n* = 35)	TRIT1 Amplified (*n* = 4)	*p* Value *
**Age years** [median (range)]	65 (49–84)	65 (49–84)	65 (55–71)	
**Gender**				
Female	8 (20.5%)	7 (20.0%)	1 (25.0%)	1.000
Male	31 (79.5%)	28 (80.0%)	3 (75.0%)	
**Smoker**				
Yes	33 (84.6%)	29 (82.9%)	4 (100.0%)	1.000
No	3 (7.7%)	3 (8.6%)	0 (0.0%)	
Unknown	3 (7.7%)	3 (8.6%)	0 (0.0%)	
**Stage**				
I	10 (25.6%)	7 (20.0%)	3 (75.0%)	0.098
II	4 (10.3%)	4 (11.4%)	0 (0.0%)	
III	11 (28.2%)	10 (28.6%)	1 (25.0%)	
IV	14 (35.9%)	14 (40.0%)	0 (0.0%)	
**Type of clinical disease**				
Localized	18 (46.2%)	15 (42.9%)	3 (75.0%)	0.318
Extensive	21 (53.8%)	20 (57.1%)	1 (25.0%)	

* *p*-value represents Fisher’s exact test or *X*^2^.

## Data Availability

DMS273 shRNA-scramble and shRNA TRIT1 RNA-seq data have been deposited in the Sequence Read Archive repository (SRA) under project code PRJNA692378. Data can be accessed through the following web address: https://www.ncbi.nlm.nih.gov/bioproject/PRJNA692378 (accessed on 13 April 2021).

## Supplementary Materials

The following are available online at https://www.mdpi.com/article/10.3390/cancers13081869/s1, Dataset S1: Methylation and Copy Number dataset for the TRIT1 and CDK5RAP1 genes in the cell lines included in the Sanger dataset (Iorio et al., Cell, 2016) and the Broad Institute Cancer Cell Line Encyclopedia (CCLE). Methylation data (orange header) includes the CpG sites interrogated with the HumanMethylation450 bead array (green = unmethylated; red = methylated), including the annotation of the CpGs in terms of chromosome (CHR), location (MAPINFO), gene (UCSC REFGENE NAME), location within the gene (USCS REFGENE GROUP) and relation to CpG Island. Copy Number data (blue header) is derived from Affymetrix Genome-Wide Human SNP Array 6.0, and regions with focal copy number alterations were identified using Genomic Identification of Significant Targets in Cancer (GISTIC) algorithm. All genomic locations are based on GRCh37/hg19., Figure S1: TRIT1 expression in SCLC cell lines assessed by immunocytochemistry, Figure S2: Effect of TRIT1 Copy Number (CN) status in DMOG (dimethyloxalylglycine) sensitivity, Figure S3: The uncropped Western blots, Table S1: mRNAs with differences between shRNA-scramble and shRNA TRIT1 depleted DMS-273 cells.
