# Supplementary material for: Gene Amplification-Associated Overexpression of the Selenoprotein tRNA Enzyme TRIT1 Confers Sensitivity to Arsenic Trioxide in Small-Cell Lung Cancer
Source: Cancers (Basel). 2021 Apr 14;13(8):1869. doi: 10.3390/cancers13081869 (PMC8070726; doi:10.3390/cancers13081869)
Supplement: Supplementary file 1 [file cancers-13-01869-s001.zip › cancers-1133923-supplementary/cancers-1133923-supplementary.pdf]

cancers

Supplementary Materials: Gene Amplification-Associated Overexpression of the Selenoprotein tRNA Enzyme TRIT1 Confers Sensitivity to Arsenic Trioxide in Small-Cell Lung Cancer

Laia Coll-SanMartin, Veronica Davalos, David Piñeyro, Margalida Rosselló-Tortella, Alberto Bueno-Costa, Fernando Setien, Alberto Villanueva, Isabel Granada, Neus Ruiz-Xiviller, Annika Kotter, Mark Helm, Jun Yokota, Reika Kawabata-Iwakawa, Takashi Kohno and Manel Esteller

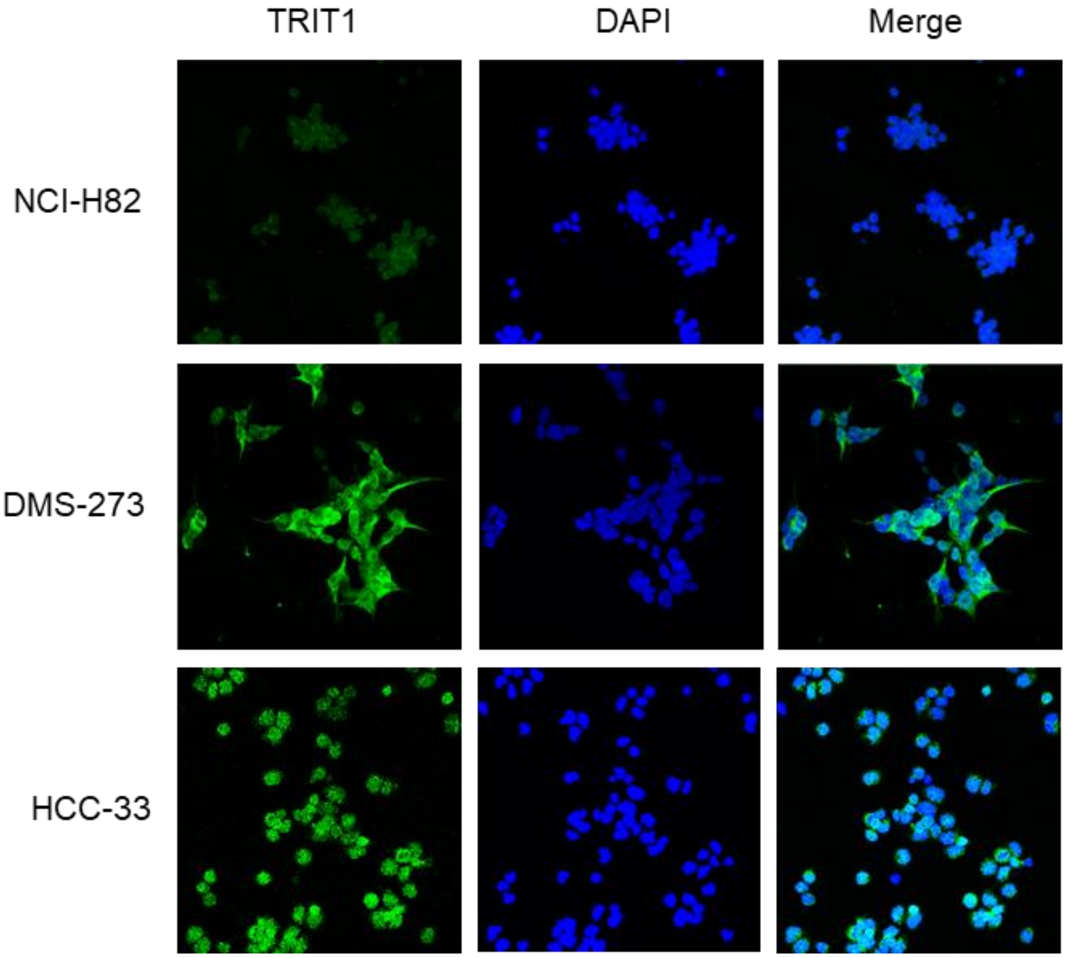

Figure S1. TRIT1 expression in SCLC cell lines assessed by immunocytochemistry. Cells were seeded on Shi-fix cover-slips (SB-Shifix50, Shikar Biotech) in a 12 wells-plate. After 30 minutes in culture to allow cell attachment, unbound cells were washed with PBS. Next, cells were fixed with 4% paraformaldehyde during 15 minutes, washed with PBS and permeabilizated with Triton X100 0.5% (Sigma Aldrich) during 10 minutes. After blocking with 5% BSA (A7906, Sigma Aldrich) diluted in PBS during 2 hours, cells were incubated overnight at 4 °C with TRIT1 antibody (NBP2-20727, Novus Biologicals, 1:200 dilution in 5% BSA in PBS). Afterwards, cover-slips were washed three times with PBS and incubated with Alexa Fluor 488 Donkey anti-Rabbit (A21206, Thermo Scientific, 1:5000 in 5% BSA in PBS). Finally, cells were washed, counterstained with DAPI and analyzed under a confocal microscope (Leica TCS SPE).

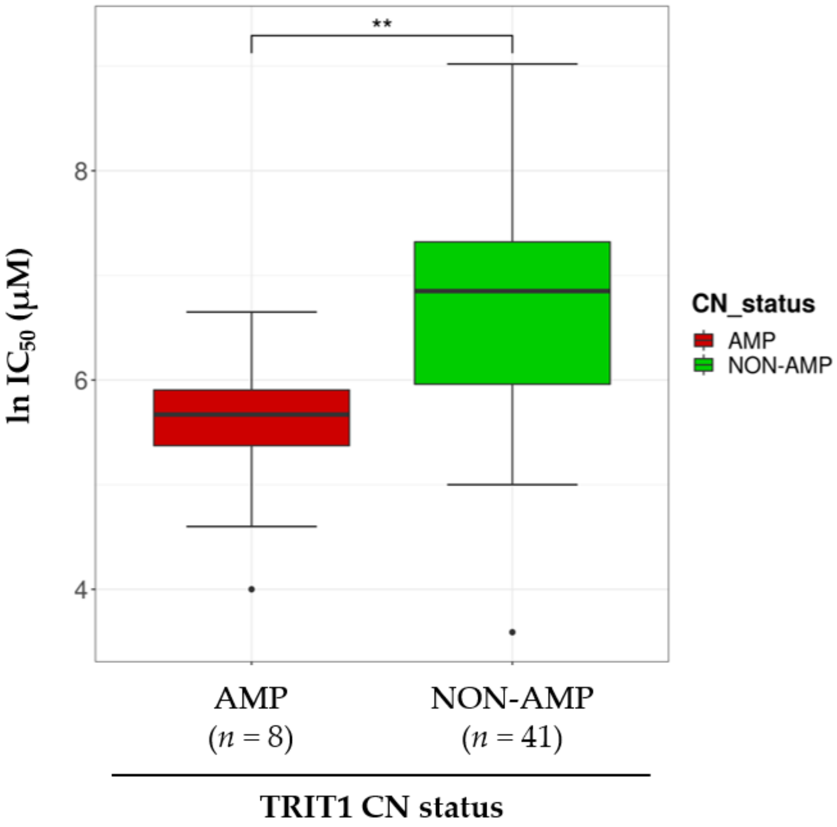

Figure S2. Effect of TRIT1 Copy Number (CN) status in DMOG (dimethyloxalylglycine) sensitivity. Natural logarithm (ln) of the half-maximal inhibitory µM concentrations (ln IC_{50}) were collected from Iorio et al. (Cell, 2016) and tested for statistical differences between TRIT1 amplified (AMP, 8 cell lines) vs TRIT1 non-amplified cell lines (NON-AMP, 41 cell lines). From the 60 cell lines with available copy number, 11 were excluded (3 AMP and 8 NON-AMP) as IC50 data was not available for DMOG. Statistical significance was assessed using Wilcoxon rank sum test with continuity correction as implemented in R statistical language (v.4.0.4). Boxplot representation was created using ggplot2 (v.3.3.3) and ggsignif (v.0.6.1) R packages. ** *p* value = 0.006.

Figure 1. **F.**

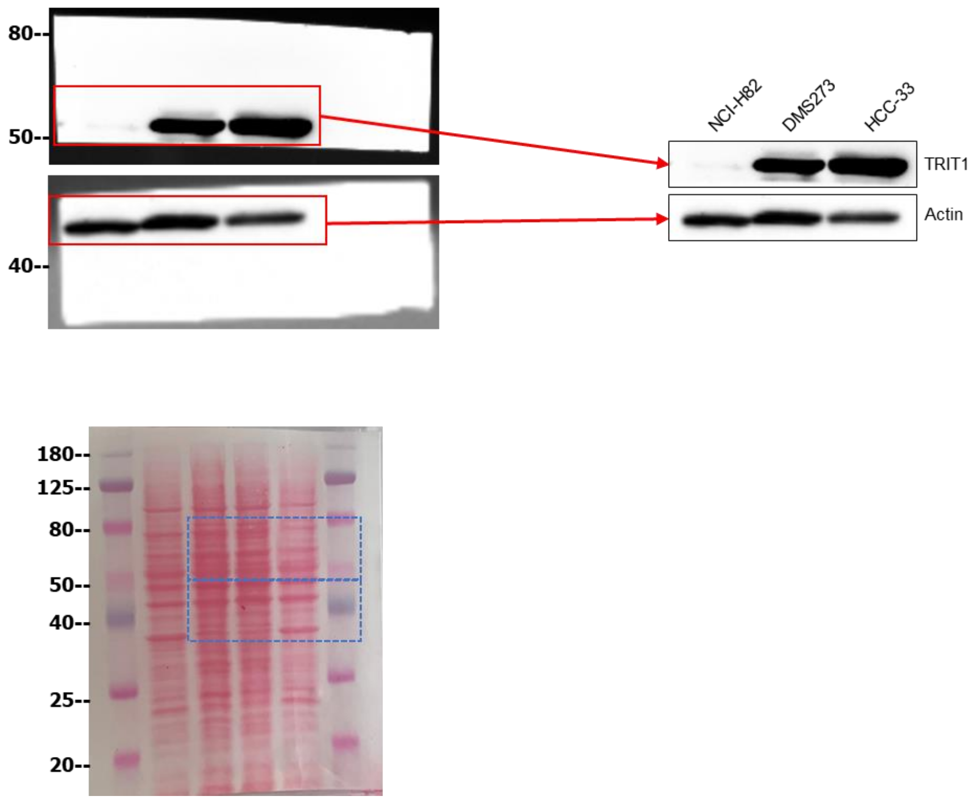

Figure 2. **A**

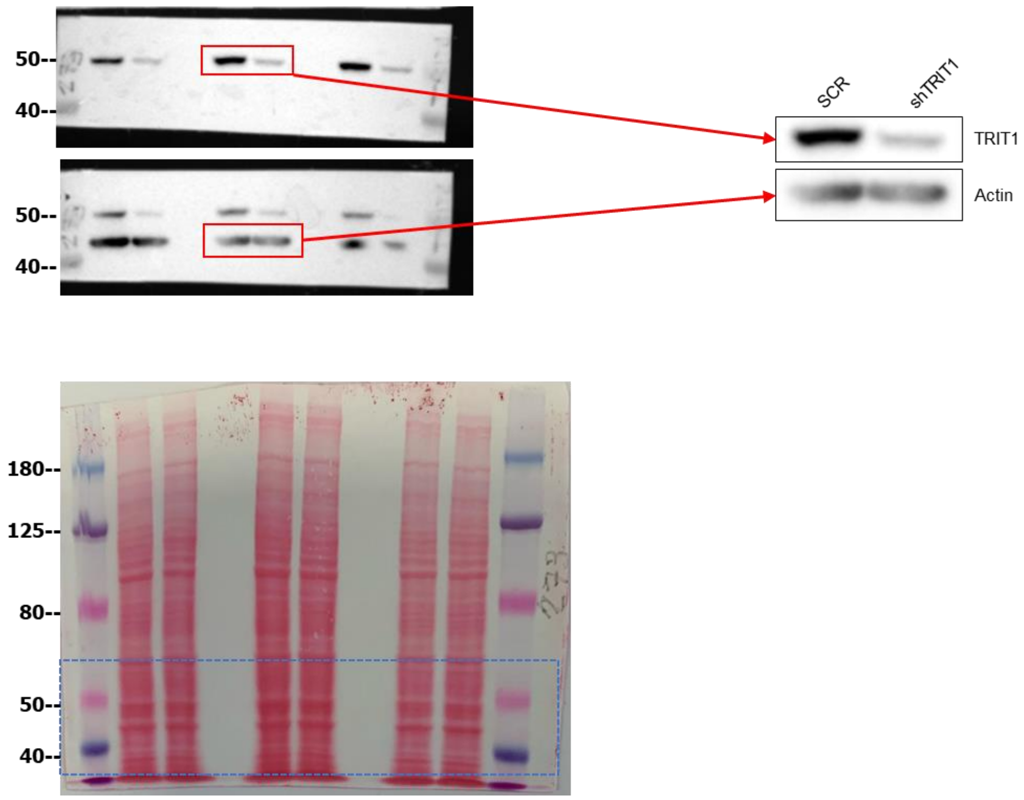

Figure S3. The uncropped Western blots.
